# Life-course leisure-time physical activity trajectories in relation to health-related behaviors in adulthood: the Cardiovascular Risk in Young Finns study

**DOI:** 10.1186/s12889-021-10554-w

**Published:** 2021-03-19

**Authors:** Irinja Lounassalo, Mirja Hirvensalo, Sanna Palomäki, Kasper Salin, Asko Tolvanen, Katja Pahkala, Suvi Rovio, Mikael Fogelholm, Xiaolin Yang, Nina Hutri-Kähönen, Olli T. Raitakari, Tuija H. Tammelin

**Affiliations:** 1 Faculty of Sport and Health Sciences, University of Jyväskylä, P.O. Box 35, FI-40014 Jyväskylä, Finland; 2 Methodology Center for Human Sciences, University of Jyväskylä, Jyväskylä, Finland; 3 Research Centre of Applied and Preventive Cardiovascular Medicine, University of Turku, Turku, Finland; 4 Centre for Population Health Research, University of Turku and Turku University Hospital, Turku, Finland; 5 Paavo Nurmi Centre, Sports & Exercise Medicine Unit, Department of Physical Activity and Health, University of Turku, Turku, Finland; 6 Department of Food and Nutrition, University of Helsinki, Helsinki, Finland; 7 LIKES Research Centre for Physical Activity and Health, Jyväskylä, Finland; 8 Department of Pediatrics, Tampere University and Tampere University Hospital, Tampere, Finland; 9 Department of Clinical Physiology and Nuclear Medicine, Turku University Hospital, Turku, Finland

**Keywords:** Physical activity, Diet, Sleep, Alcohol, Binge drinking, Smoking, Screen time, Longitudinal, Trajectory, Life-course

## Abstract

**Background:**

Evidence on whether leisure-time physical activity (LTPA) facilitates individuals’ adoption of multiple healthy behaviors remains scarce. This study investigated the associations of diverse longitudinal LTPA trajectories from childhood to adulthood with diet, screen time, smoking, binge drinking, sleep difficulties, and sleep duration in adulthood.

**Methods:**

Data were drawn from the Cardiovascular Risk in Young Finns Study. Participants were aged 9–18 years (*N* = 3553; 51% females) in 1980 and 33–49 years at the latest follow-up in 2011. The LTPA trajectories were identified using a latent profile analysis. Differences in self-reported health-related behaviors across the LTPA trajectories were studied separately for women and men by using the Bolck-Croon-Hagenaars approach. Models were adjusted for age, body mass index, education level, marital status, total energy intake and previous corresponding behaviors.

**Results:**

Persistently active, persistently low-active, decreasingly and increasingly active trajectories were identified in both genders and an additional inactive trajectory for women. After adjusting the models with the above-mentioned covariates, the inactive women had an unhealthier diet than the women in the other trajectories (*p* <  0.01; effect size (ES) > 0.50). The low-active men followed an unhealthier diet than the persistently and increasingly active men (*p* <  0.01; ES > 0.50). Compared to their inactive and low-active peers, smoking frequency was lower in the increasingly active women and men (*p* <  0.01; ES > 0.20) and persistently active men (*p* <  0.05; ES > 0.20). The increasingly active men reported lower screen time than the low-active (*p* <  0.001; ES > 0.50) and persistently active (*p* <  0.05; ES > 0.20) men. The increasingly and persistently active women reported fewer sleep difficulties than the inactive (*p* <  0.001; ES > 0.80) and low-active (*p* <  0.05; ES > 0.50 and > 0.80, respectively) women. Sleep duration and binge drinking were not associated with the LTPA trajectories in either gender, nor were sleep difficulties in men and screen time in women.

**Conclusions:**

Not only persistently higher LTPA but also an increasing tendency to engage in LTPA after childhood/adolescence were associated with healthier diet and lower smoking frequency in both genders, having less sleep difficulties in women and lower screen time in increasingly active men. Inactivity and low activity were associated with the accumulation of several unhealthy behaviors in adulthood. Associations were stronger in women.

**Supplementary Information:**

The online version contains supplementary material available at 10.1186/s12889-021-10554-w.

## Background

Non-communicable diseases, such as cardiovascular disease, diabetes mellitus, and several cancers, cause over 70% of deaths worldwide with unhealthy behaviors usually causing these diseases [1]. Physical activity (PA), dietary behavior, smoking and consuming alcohol are traditionally seen as the major four behaviors impacting health [2]. Lately, sleeping and sedentary behavior have been identified as important additional behaviors impacting it as well [3–6]. Physical inactivity, unhealthy diet, regular smoking, and binge drinking [7], shortened [4] and prolonged sleep duration [3, 4], insomnia [5], and sedentary behavior [6, 8] (e.g., prolonged television viewing [9] and total screen time [10]) have been found to be associated with a higher risk of non-communicable diseases and mortality.

Behavioral risk factors tend to accumulate to same individuals which may have synergistic effects on health [11]. A study examining smoking, alcohol intake, diet, PA, television viewing, and sleep together showed that a combination of multiple health-compromising behaviors was strongly associated with cardiovascular disease mortality and all-cause mortality [12] while a combination of multiple health-enhancing behaviors have been found to decrease the relative risk for all-cause mortality [13]. Thus, promoting the adoption of multiple healthy behaviors is essential for improving public health.

Changing one behavior may translate into changing other behavior as well, especially if both behaviors are health-enhancing or health-risky [14]. Previous evidence suggests that PA might play an important role for adopting to multiple healthy lifestyle factors, e.g. eating healthier [15, 16]. A longitudinal study found that adults who increased their PA also improved their diet when compared to their decreasingly active peers [17], whereas low PA was found to associate with higher risk of sleep problems and incident short sleep time among middle-aged and older adults [18]. Additionally, physical inactivity in adolescence predicted smoking [19] and weekly alcohol intoxication in young adulthood, especially in women [20]. Our previous longitudinal studies showed that persistently physically active adult women watched television less than low-active women [21], leisure-time physical activity (LTPA) evolved in tandem with fruit and vegetable consumption from childhood to adulthood [22], persistent inactivity was associated with smoking [23, 24] whereas, in contrast, persistent higher LTPA was associated with regular alcohol drinking [23].

However, previous research has mainly focused on the association of PA or LTPA with single health behavior. Moreover, longitudinal studies on whether a specific LTPA life-course developmental pathway facilitates the adoption of several other health-enhancing behaviors in adulthood are lacking. This study aimed to investigate whether cross-sectionally measured health-related behaviors in adulthood – including diet, screen time, smoking, binge drinking, sleep difficulties, and sleep duration – differ by LTPA pathways measured from childhood to adulthood in men and women. LTPA was defined as participation in PA and sport by querying intensity, duration and frequency of PA, and participation in organized sports. Occupational PA was not queried. Data-driven trajectory modeling [25] was chosen as the method for identifying LTPA pathways, as trajectories can yield novel information on the complexity of PA behavior [26].

## Methods

### Study design and participants

The data were drawn from the ongoing population-based longitudinal Cardiovascular Risk in Young Finns Study (YFS). The study involves five Finnish university cities (Helsinki, Kuopio, Oulu, Tampere, and Turku) and their rural surroundings. Participants with different socioeconomic backgrounds were randomly selected from the population register of these areas to produce a representative sample of Finnish children and adolescents [27]. In 1980, 3596 children and adolescents (boys and girls aged 3–18 years; 83% of those invited) participated in the baseline study. Follow-up studies have thus far been conducted in 1983, 1986, 1989, 1992, 2001, 2007 and 2011. The study sample comprised six cohorts aged 3, 6, 9, 12, 15 and 18 years at baseline and thus 33–49 years at the 2011 follow-up. The representativeness of the study population has been studied by comparing the baseline (1980) characteristics between the sample of the year 2001 and those lost to follow-up [27]. The results showed that participants were older and more often females than non-participants. No significant differences between the non-participants and participants were observed in LTPA, body mass index (BMI), or parental years of education. A more detailed description of the YFS protocol, attrition analysis and reasons for non-participation have been published previously [27].

### Measurements

#### Life-course leisure-time physical activity

LTPA was parent-reported before turning 9 years of age and self-reported thereafter. To avoid biased results, parent-reported data were excluded from the present study. Participants with at least one measurement of self-reported LTPA (*N* = 3553) were included in the present study (see more in [Supplementary file 1]). Self-reported LTPA was treated as the exposure variable for other adulthood health-related behaviors. In the years 1980–1989, the LTPA questionnaire comprised items on the frequency and intensity of PA, habitual ways of spending leisure-time, and participation in sports-club training and sport competitions. After 1989, the questionnaire was slightly modified, as LT﻿PA differs between school-aged children and adults. In the years 1992–2011, the LTPA questionnaire comprised items on the frequency and intensity of PA, frequency of vigorous PA, hours spent in vigorous PA, average duration of a PA session, and participation in organized PA. Occupational PA was not queried. Responses on each item were first recoded into three categories (1 = irregular activity / low activity / inactivity; 2 = regular weekly activity / moderate activity; 3 = regular daily activity / vigorous activity) and scores were then summed to create a LTPA index ranging from 5 to 15 [28]. The development of the index has been reported in detail elsewhere (see Table S1 and S2 in [23]).

In the YFS, the original aim of the LTPA index was not to measure the absolute amount or exposure to LTPA but rather to rank the participants according to their LTPA level. Previously conducted validation analyses show that the LTPA questionnaire is an acceptably valid subjective measure of LTPA [29–32]. Correlations between the LTPA index and indicators of exercise capacity (hypothetical maximal workload sustainable for 6 min) have been studied in subsamples of participants and found to be significant with medium effect sizes in childhood (girls: *r* = 0.39; boys: *r* = 0.33) and medium to large effect sizes in adulthood (women: *r* = 0.49; men: *r* = 0.53) [30]. The index also correlated with the total volume of movement assessed with accelerometers, including frequency, intensity, and duration of activity bouts (*r* = 0.26–0.45) [31], and with 7-day pedometer data on total steps and aerobic steps (*r* = 0.25–0.31) [32] with the effect sizes ranging from small to medium. In 1980, internal consistency coefficients, as indicators of reliability, varied from 0.44 to 0.69 in females and 0.49 to 0.76 in males, and in 2001, the corresponding values were 0.59 to 0.85 and 0.74 to 0.85 [30].

#### Measurements of health-related behaviors in adulthood

In 2011, the adulthood health-related behaviors assessed with self-reports were diet, screen time, smoking, binge drinking, sleep difficulties and sleep duration. These behaviors were treated as the outcome variables for the LTPA trajectories.

Diet was assessed with a validated Finnish self-administered food frequency questionnaire [33]. Participants reported the frequencies of their habitually consumed food and drink items. Average daily food intake was converted into grams by using the Finnish National Food Composition Database (Fineli®). A sum index indicating a healthy diet was created. It included foods considered healthy (whole grain products; fruits excluding fruit juice; vegetables and legumes excluding potatoes; fish; vegetable fats and oils) or unhealthy (red meat and meat products; sugared beverages; fried and deep-fried potatoes; desserts including budding, ice cream, biscuits, sweet pastry, chocolate and candy). Participants were first divided into quartiles (Q) based on their mean daily intake scores for each food item: the higher the consumption of healthy foods, the higher the score (Q1 = 0; Q2 = 1; Q3 = 2; Q4 = 3 points) and the higher the consumption of unhealthy foods, the lower the score (Q1 = 3; Q2 = 2; Q3 = 1; Q4 = 0 points). Points were then summed to create a diet index ranging from 0 to 27: the higher the value, the healthier the participant’s diet.

Television viewing time and computer use were assessed with two questions: “How many hours per day on average day do you spend: 1) watching television and videos at home, and 2) using a computer at home?” Mean daily television and computer times were calculated separately ([(5 × weekday) + (2 × weekend)] / 7) after which the mean values of the two sedentary behaviors were summed to form an index of daily screen time during leisure (hours/day).

The categories for current smoking status were: (1) non-smoker (never smoked), (2) former smoker (on strike or stopped smoking), (3) occasional smoker (less than once a week), and (4) regular smoker (once or more a week). Binge drinking was determined as consuming six or more alcoholic drinks on a single occasion. The categories for binge drinking frequency were: (1) six times a year or less, (2) one to three times a month, (3) once a week, and (4) twice a week or more often.

Sleep difficulties were assessed with Jenkins’s scale which has proven valid and reliable [34, 35]. The scale comprises four items on sleep difficulties corresponding to the insomnia symptoms listed in the Diagnostic and Statistics Manual of Mental Disorders [36]. The four items assess problems in falling asleep, awakenings during sleep, difficulties staying asleep (including too-early awakenings), and feelings of tiredness after a normal night’s sleep [34]. For participants who reported more than one symptom, their most frequent symptom was recorded as their degree of sleep difficulty. Sleep difficulty was categorized as 1) no sleep difficulty (≤ 1 night a week), 2) moderate sleep difficulty (2–4 nights a week), or 3) severe sleep difficulty (5–7 nights a week).

Hours of usual sleep duration per night were self-reported within half an hour accuracy. The recommended duration of sleep for young adults and adults is 7–9 h per night [37]. The results were reported as probabilities for meeting the sleep duration recommendation. A binary variable was created with 1 = meeting and 0 = not meeting the recommendation.

#### Covariates

Age, body mass index (BMI), level of education and marital status were elicited in 2011 and used as covariates, as they may affect health behaviors [38–41]. Total energy intake was used as a covariate for dietary behavior. Also, health behaviors in childhood, adolescence or young adulthood were used as covariates for the corresponding outcome health behaviors in adulthood, since previous behavior predicts similar behavior in the future [42–44]. A detailed description of the covariates can be found in supplementary material [Supplementary file 2].

### Statistical analysis

Descriptive statistics were calculated by using IBM SPSS Statistics for Windows, version 24.0 (IBM Corp. Armonk, NY, USA) and expressed as means and standard deviations or as percentages. Differences in the study variables between women and men were tested by using an independent-samples t-test.

Further modeling was performed with Mplus, version 8.0 [45]. All analyses were performed separately for women and men due to previously observed sex differences in LTPA [24] and other health-related variables (Table 1). The specific LTPA trajectories for women and men used in the current study were earlier identified using latent profile analysis. For details on the statistical modeling, see supplementary material [Supplementary file 1] and previous papers ([22, 24]). In the trajectory modeling, missing data were assumed to be missing at random. Model parameters were estimated by using the full information maximum likelihood method with robust standard errors, which enabled use of all available data.

**Table 1:** Descriptive statistics of the study sample and baseline LTPA in 1980

	Women (*N* = 1813)		Men (*N* = 1740)		
	Mean (SD) / %	*n*	Mean (SD) / %	*n*	*p* ^a^
LTPA in 1980 (index value, range 5–14)	8.6 (1.6)	1200	9.5 (1.9)	1151	< 0.001
LTPA in 2011 (index value, range 5–15)	9.1 (1.9)	1072	8.9 (1.9)	852	0.016
Body mass index in 2011 (kg/m2)	26.1 (5.5)	1118	27.0 (4.4)	931	< 0.001
Total energy intake in 2011 (kcal / day)	2090.0 (602.9)	998	2623.8 (775.6)	734	< 0.001
Education in 2011 (years)	15.7 (3.4)	1101	14.9 (3.8)	884	< 0.001
Marital status in 2011:					
Married / in registered relationship / cohabiting	77.5%	860	79.9%	712	
Unmarried / divorced / separated / widowed	22.5%	249	20.1%	179	
Healthy diet index in 1989 (range 9–54)	36.1 (4.4)	1349	34.4 (4.6)	1078	< 0.001
Healthy diet index in 2011 (range 0–27)	14.9 (4.0)	1014	11.9 (4.0)	747	< 0.001
Screen time during leisure in 2001 (h / day)	1.9 (1.2)	1353	2.2 (1.4)	1096	< 0.001
Screen time during leisure in 2011 (h / day)	2.7 (1.5)	1095	3.1 (1.7)	875	< 0.001
Smoking status in 1989 (range 1-4)	2.0 (1.2)	1471	2.2 (1.3)	1234	< 0.001
Non-smoker	50.4%	742	48.1%	594	
Former smoker	20.8%	306	14.3%	177	
Occasional smoker	6.5%	96	6.7%	83	
Regular smoker	22.2%	327	30.8%	380	
Smoking status in 2011 (range 1-4)	1.8 (1.1)	1110	2.0 (1.2)	889	< 0.001
Non-smoker	55.9%	621	44.7%	397	
Former smoker	24.6%	273	28.9%	257	
Occasional smoker	3.7%	41	5.2%	46	
Regular smoker	15.8%	175	21.3%	189	
Occasions of binge drinking in 1989 (range 1-5)	2.6 (1.6)	1437	2.9 (1.7)	1217	< 0.001
None	40.3%	579	39.6%	482	
One time	9.0%	129	5.8%	70	
Two to three times	17.9%	257	10.5%	128	
Four to ten times	15.4%	221	12.6%	153	
More than 10 occasions	17.5%	251	31.6%	384	
Binge drinking in 2011 (consumed > 6 drinks on one occasion; range 1-4)	1.4 (0.7)	1099	1.9 (1.0)	877	< 0.001
Six times a year or less	75.3%	827	48.0%	421	
One to three times a month	16.7%	184	27.9%	245	
Once a week	4.5%	49	14.4%	126	
Twice a week or more often	3.5%	39	9.7%	85	
Feeling fatigue in 1986 (range 1-4) ^b^	2.8 (0.9)	691	2.4 (1.0)	554	< 0.001
Rarely or never	10.0%	69	23.5%	130	
Once a month	27.4%	189	29.2%	162	
Once a week	40.5%	280	35.0%	194	
Daily	22.1%	153	12.3%	68	
Sleep difficulties in 2011 (range 1-3)	1.7 (0.8)	1112	1.5 (0.7)	894	< 0.001
No sleep difficulty	51.4%	572	61.4%	549	
Moderate sleep difficulty	28.4%	316	24.8%	222	
Severe sleep difficulty	20.1%	224	13.8%	123	
Sleeping time in 1986 (h/night)^b^	7.8 (0.9)	694	7.7 (0.9)	559	0.082
Less than seven hours per night	5.8%	40	8.6%	48	
Recommended 7–9 h per night	90.6%	629	89.1%	498	
More than nine hours per night	3.6%	25	2.3%	13	
Sleeping time in 2011 (h/night)	7.4 (1.0)	1111	7.1 (0.9)	892	< 0.001
Less than seven hours per night	18.8%	209	27.7%	247	
Recommended 7–9 h per night	78.4%	871	71.3%	636	
More than nine hours per night	2.8%	31	1.0%	9	

*SD* standard deviation; *LTPA* leisure-time physical activity. ^a^ *p*-value for sex difference (t-test). ^b^ Assessed only from the 18, 21 and 24 year-old participants

In the current study, mean differences in the diet index, screen time, smoking status, binge drinking, sleep difficulties, and sleep duration were studied across the LTPA trajectories with the 2011 data. The Bolck-Croon-Hagenaars (BCH) approach was utilized for this purpose [46–48]. In the BCH approach, the model estimates for the latent classes (i.e., LTPA trajectories) are not affected by the auxiliary variables (i.e., diet, screen time, smoking, binge drinking, sleep difficulties, and sleep duration), thereby preventing changes in LTPA trajectory class membership [48]. BCH weights were saved from the latent profile analysis run with the previously identified optimal number of LTPA trajectories. BCH weights are group-specific weights computed for each participant during the latent profile model estimation. BCH weights were used as training data and a multiple group regression model was estimated.

In the first step (model 1), the mean values of the health-related behaviors studied across the LTPA trajectory classes were unadjusted. To interpret mean differences required a Wald test *p*-value of <.05. In the second step (model 2), the mean values of the health-related behaviors were adjusted for age, BMI, education level and marital status. Model 2 was also adjusted for total energy intake when studying the mean diet index across the LTPA trajectories. In the third step (model 3), the mean values of the health-related behaviors were additionally adjusted with those of the previous corresponding health-related behaviors. When adjusting models 2 and 3, the mean values of the health behaviors were regressed on the above-mentioned covariates for the distinct LTPA trajectories. Differences in the regression intercepts (i.e., adjusted means of the health-related behaviors) were studied across the trajectory classes. Models were adjusted using standardized values of the covariates.

Cohen’s d [49] and Cohen’s h [50] were calculated for providing measure of effect concerning the mean differences of the health-related behaviors across the LTPA trajectory classes. Effect sizes were interpreted as small (≥0.20), medium (≥0.50), and large (≥0.80). Cohen’s d measures the difference between two means divided by a standard deviation and Cohen’s h is a measure of distance between two proportions [49, 50]. Cohen’s d was calculated by using variances from the unadjusted models and intercepts (means) from the adjusted models 2 and 3. Cohen’s h was calculated for probabilities of following sleep recommendations across the LTPA trajectory classes from the adjusted models 2 and 3.

The sample sizes dropped, especially in model 3. To verify the robustness of the models, sensitivity analyses were performed. Unadjusted analyses were performed for the same sample as in model 3 and then compared to the original results of model 1. The results of the sensitivity analyses closely resembled those of the main analyses. The main differences were in the associations between the LTPA trajectories and sleep difficulties in women: two additional significant associations were observed (*p* <  0.05). In 1986, the sleep difficulties variables were only assessed in the three oldest age cohorts. Thus, sleep difficulties in women were assessed at a higher mean age in model 3 than in model 1. However, model 3 was considered to be adequately reliable as it included all the available data with the relevant adjustments.

### Quality assessment

To enhance reporting quality, this study was conducted according to extension of the Strengthening the Reporting of Observational Studies in Epidemiology guidelines for nutritional epidemiology (STROBE-nut) [51] [Supplementary file 3]. The Guidelines for Reporting on Latent Trajectory Studies (GRoLTS) checklist [52] was applied to enhance the quality of the latent profile analyses used (see previous study [22]).

## Results

### Participants and their characteristics

The sample size of the study was 3553 (51% females). All eight LTPA measurements had been completed by 4% of the participants, seven by 8%, six by 16%, five by 23%, four by 18%, three by 15%, two by 10% and one by 6%. Descriptive information on the participants is shown in Table 1.

### Life-course leisure-time physical activity trajectories

**Fig. 1:**
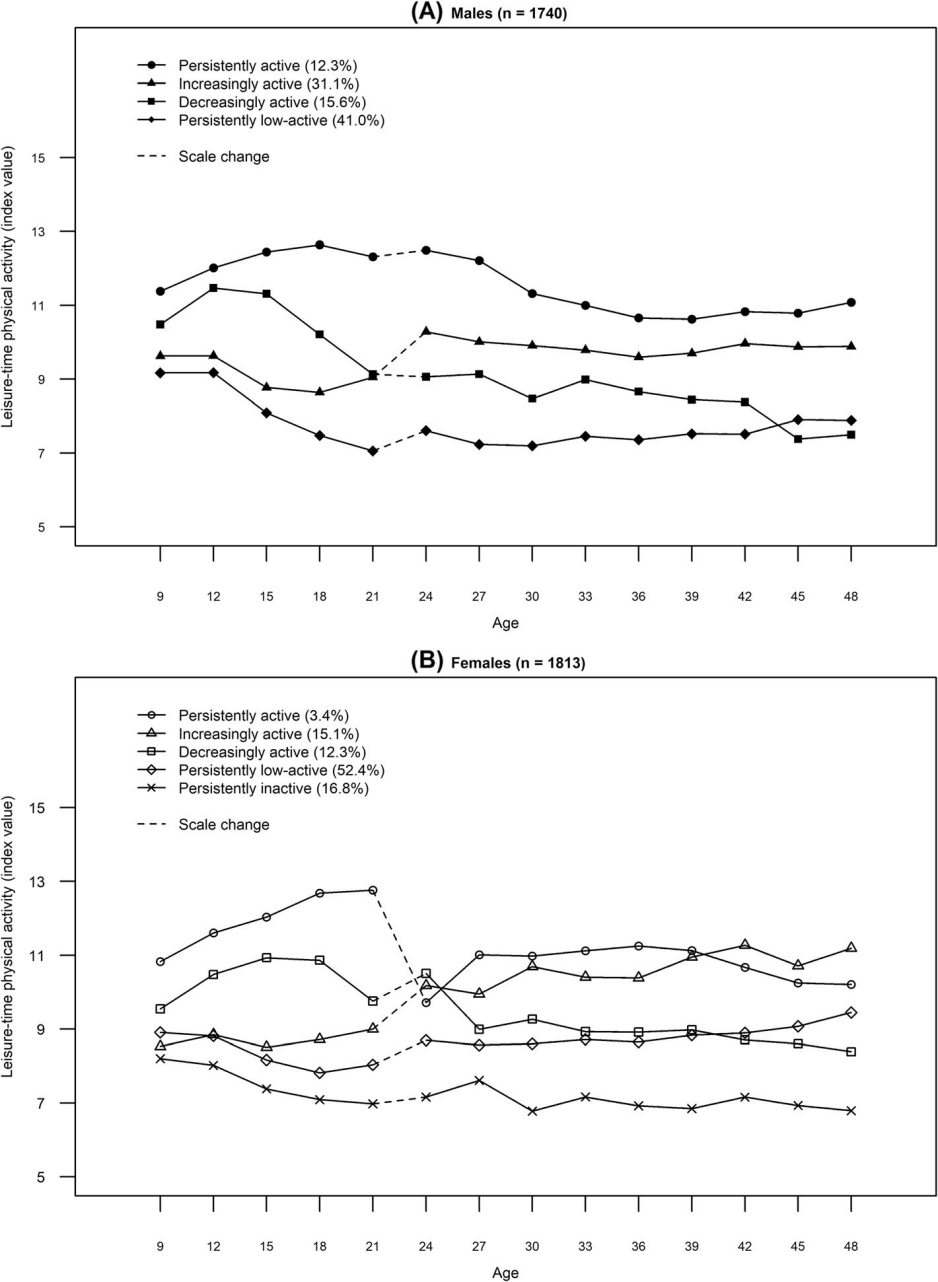
Leisure-time physical activity trajectories for males (**a**) and females (**b**) (also published in [22, 24])

Four similar LTPA trajectories were identified for both men and women from childhood to adulthood: persistently low-active, decreasingly active, increasingly active, and persistently active (Fig. 1a and b). An additional trajectory labeled inactivity was identified in women (Fig. 1b). The selection of the final number of trajectory classes has previously been reported in detail [22]. While the present trajectories were the same in shape and number as those previously identified, membership of the persistently low-active and decreasingly active trajectories had increased by 0.1% in males. More detailed description of the shapes of the trajectories can be found in the supplementary material [Supplementary file 1].

### Adulthood health-related behaviors across the leisure-time physical activity trajectories

#### Dietary behavior

**Fig. 2:**
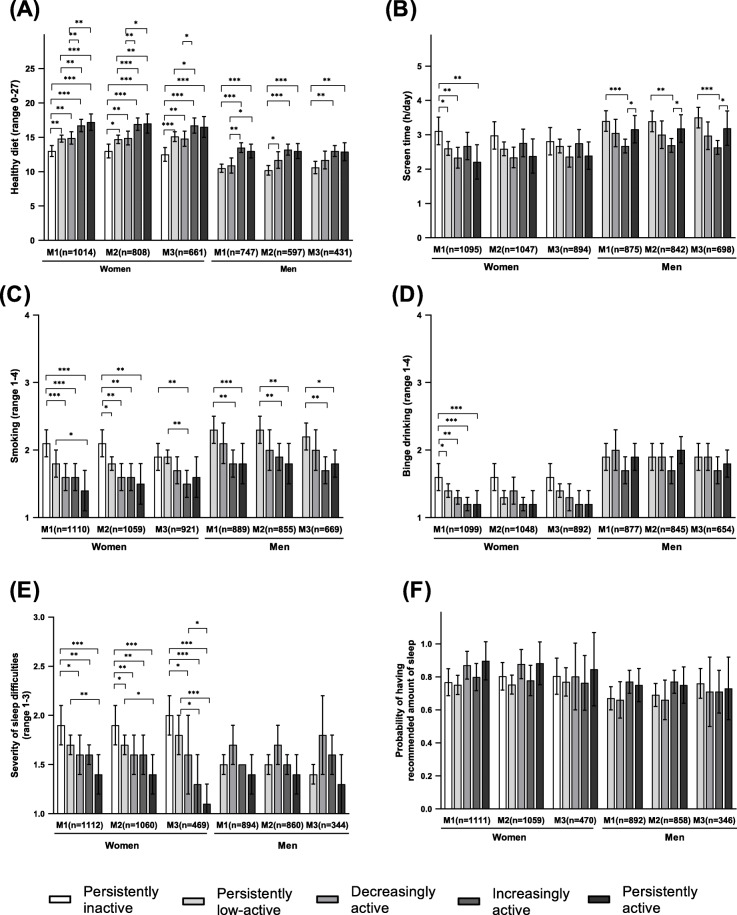
Mean values and 95% confidence intervals for each health behavior (**a** = healthy diet; **b** = screen time; **c** = smoking; **d** = binge drinking; **e** = severity of sleep difficulties; **f** = probability of having recommended amount of sleep) across the leisure-time physical activity trajectories in the unadjusted model (M1) and adjusted models (M2 and M3) among women and men aged 33 to 49 years. M2 was adjusted for age, BMI, education, and marital status and diet additionally adjusted for total energy intake. In M3, each behavior was additionally adjusted for the corresponding earlier behavior: diet assessed in 1989, screen time in 2001, binge drinking in 1989, smoking in 1989, fatigue in 1986 and sleep duration in 1986. Significant mean differences in each health behavior across the LTPA trajectory classes in each model (M1–3) are marked with an asterisk (* = *p* < 0.05; ** = *p* < 0.01; *** = *p* < 0.001)

After all adjustments, the highest mean values of the diet index (range 0–27) were observed in the increasingly active (mean 16.8) and the lowest in the persistently inactive trajectory (12.6) among women while men in the persistently active trajectory had the highest mean values (13.0) and men in the persistently low-active one the lowest (10.7) (Fig. 2a, model 3). Women in the persistently inactive trajectory group had an unhealthier diet in adulthood than those in the other trajectory groups (Fig. 2a, models 1–3). The effect sizes were large between the persistently inactive and increasingly active and, also, the persistently inactive and active women. All effect sizes (Cohen’s *d* and *h*) can be found in the supplementary material [Supplementary file 4]. The increasingly active women additionally had a healthier diet than their persistently low-active and decreasingly active peers. The persistently active women had a higher mean diet index than their low-active and decreasingly active peers (models 1–2), but these associations were attenuated after adjustment for previous dietary behavior (model 3). Among men, those in the persistently low-active trajectory had a lower mean diet index than their persistently and increasingly active peers (Fig. 2a, models 1–3). The decreasingly active men had an unhealthier diet than peers in the increasingly and persistently active trajectories (model 1) and a healthier diet than the persistently low-active men (model 2); however, these significant associations disappeared after full adjustments (model 3).

#### Screen time

After fully adjusting the models (Fig. 2b, model 3), the highest mean leisure screen time was observed among the persistently inactive women (mean 2.8 h / day) and low-active men (3.5). The lowest mean leisure screen time was observed in the decreasingly active women (2.3) and the increasingly active men (2.6). Before adjustments, women in the inactive trajectory had higher leisure screen time than peers in the persistently, decreasingly and low-active trajectories (Fig. 2b, model 1). After adjustments, these significant associations disappeared. In contrast, mean differences in leisure screen time across the LTPA trajectories in men remained after adjustments (Fig. 2b, models 1–3). Men in the increasingly active trajectory had lower mean leisure screen time than their persistently low-active and persistently active peers.

#### Smoking

The mean values of smoking (range 1–4) were highest in the persistently inactive and low-active women (mean 1.9) and the persistently low-active men (2.2) whereas the increasingly active women and men had the lowest mean values of smoking (1.5 and 1.7, respectively) after full adjustments (Fig. 2c, model 3). The persistently inactive and low-active women were more frequently regular smokers than their increasingly active peers. Similarly, the persistently low-active men smoked more frequently than their persistently active and increasingly active peers (Fig. 2c, models 1–3).

#### Binge drinking

The mean values of binge drinking (range 1–4) were highest among the persistently inactive women (mean 1.6), and persistently low-active (1.9) and decreasingly active (1.9) men. The lowest mean values of binge drinking were observed in the increasingly and persistently active women (1.2) and increasingly active men (1.7) (Fig. 2d, model 3). The unadjusted analyses showed that the persistently inactive women were more frequently binge drinkers than the women in the other trajectories (Fig. 2d, model 1). After adjustments, no differences in binge drinking were found across the LTPA trajectory groups in women nor in men (Fig. 2d).

#### Sleep difficulties and sleep duration

The mean value of sleep difficulties (range 1–3) was highest in the persistently inactive trajectory of women (mean 2.0) and decreasingly active trajectory for men (1.8) while the persistently active women and men had the lowest mean values: 1.1 and 1.3, respectively (Fig. 2e, model 3). Significant mean differences in sleep difficulties between the LTPA trajectories were observed for women, but not for men (Fig. 2e). The persistently inactive women reported more sleep difficulties than their persistently, increasingly and decreasingly active peers. Moreover, the low-active women reported more sleep difficulties than their persistently (models 1–3) and increasingly active peers (model 3). No differences in the probability of having the recommended 7–9 h of sleep was observed between the LTPA groups in either gender (Fig. 2f, models 1–3).

## Discussion

This study examined how diverse pathways (i.e., trajectories) of LTPA from childhood to adulthood were associated with diet, screen time, smoking, binge drinking, sleep difficulties and sleep duration in adulthood in men and women. Higher LTPA was associated with non-smoking and following a healthier diet in both genders and with less frequent sleep difficulties in women. Men whose LTPA increased in young adulthood reported lower screen time than persistently active and low-active men. Thus, those in the persistently, and especially increasingly active, life-course trajectories had accumulated several health-enhancing behaviors. Simultaneously, those in the inactive or low-active life-course trajectories had accumulated health-compromising behaviors. This is a concern from the public health perspective, as the biggest proportion of participants, of both sexes, were in the low-active trajectories. Our results are supported by previous cross-sectional [2, 53] and longitudinal [54, 55] findings showing a clustering of health behaviors at both ends of the lifestyle spectrum: the healthy and unhealthy. Previous findings show the phenomenon being more evident in women than men [38] which was observed also in the current study with generally higher effect sizes observed in women.

The poorest diet was found in the inactive trajectory for women and the low-active trajectory for men. This supports our previous findings [22] and those of another study showing that PA and fruit and vegetable consumption, an indicator of a healthy diet, are accumulated by the same individuals [53]. Of the studied behaviors, dietary behavior in adulthood – especially in women – showed the strongest association with the life-course LTPA trajectories. Several possible mechanisms behind the association have been proposed. For example, positive experiences from exercising may improve self-esteem [56], and body-image [57], and lead to promote individuals’ self-efficacy and motivation to modify dietary habits as well [58–60]. Also, the similar barriers and motivators experienced for these two behaviors could explain the strong association. A study by Ashton et al. [61] showed that, in young men, motivators for PA and healthy eating were improving physical health, performance and physical appearance while logistic barriers (cost and access) and social factors (e.g., peer influence) were found as barriers for these two behaviours. Generally, women perceive healthy eating as more important [62, 63], experience a higher need for weight control [62], and are more health conscious [64] than men which could explain why the associations between LTPA and diet were stronger in women.

The strongest associations between LTPA trajectories and sleep difficulties were detected in the fully adjusted model for women that included only the three oldest age cohorts (42–49-year-old participants). Sleep difficulties were less prevalent among the increasingly and persistently active women than among their persistently low-active and inactive peers, while no associations were found among men. Generally, poor sleep quality is more prevalent among women than men [65]. In our sample, only 14% of men and up to 20% of women reported severe sleep difficulties in their forties. One explanation for the sex difference is probably the menopausal transition which is related to adverse changes in sleep quality [66, 67]. Previous studies on older adults have reported higher PA to be associated with maintenance of sleep sufficiency [68], lower probability of daytime sleepiness [69], and less sleep difficulties [18]. Among middle-aged adults, the findings are contradictory, some studies reporting an inverse [18, 68] and others no association between PA and sleep quality [69]. When studying adolescents, thus younger populations, no associations between PA trajectories and sleep time were found [70]. These age group-specific associations might reflect the aging-related decline in sleep quality [71] and, on the other hand, the importance of being physically active while ageing as it may improve sleep quality. Older age and female gender [69] both seemed to explain the associations we found between LTPA trajectories and sleep difficulties.

Several studies have reported an inverse association between PA and smoking: for example, smoking [72] and nicotine dependency [73] predicted lower levels of PA. Conversely, inactivity or occasional activity in adolescence predicted a higher prevalence of daily smoking in young adulthood [19]. Both our previous [23, 24] and current results corroborate those findings: LTPA development was inversely associated with smoking in adulthood for both genders – though not as strongly as it was with dietary behavior. Possible explanations for this include the clustering of positive and negative behaviors, and various psychological (e.g., depression), socio-demographic (e.g., education level), or physiological (e.g., lung capacity) factors [74]. Also, people who are physically active usually value physical fitness and strength [75], and are aware that smoking weakens the possibility to improve them.

Somewhat unexpectedly, not only the persistently low-active but also the persistently active men reported higher leisure screen time than their increasingly active counterparts. Earlier studies have found that men watch more sport on television [76], engage more in video gaming [77] and are more sensation-seeking (i.e., willing to engage in novel and intense activities) [78] than women. The persistently active trajectory might include a selected group of men seeking intense activities via participating in sports, viewing sports and playing e-sports or other video games. After full adjustments, no associations between LTPA trajectories and screen time in women were observed. Similarly, LTPA trajectories were not associated with sleeping duration nor binge drinking in either gender which differed from previous findings [18, 20].

The strengths of the current longitudinal study were its six age cohorts and several measurement points during a follow-up of over 30 years, enabling the study of the associations between life-course LTPA trajectories and several other health behaviors in adulthood. In this study, we expanded our previous research on health behaviors by including sleeping behavior, using an overall healthy diet index instead of measuring only fruit and vegetable consumption, and adding screen time to television viewing as sedentary behavior. Finally, instead of regular alcohol consumption we studied binge drinking, since this has been found to have marked effects on cardiovascular disease morbidity and mortality [79].

The study had its limitations. The use of self-reports may bias the results and lead to under- and over-reporting [80–82]. The proportion of women identified in the persistently active trajectory was small (3.4%): the association between persistent lifelong LTPA and other health behaviors among women should be confirmed in future studies. The measure of screen time did not include the use of electronic mobile devices such as smart phones or tablet computers and therefore does not cover all aspects of screen time. The models were adjusted for previous corresponding behaviors in order to ascertain whether the association between LTPA and a behavior is predicted by LTPA or by the behavior itself. This led to a lower sample size in the fully adjusted models which is why sensitivity analyses were performed in order to detect potential selection bias. Additionally, previous sleeping behavior in 1986 was assessed only from the three oldest age cohorts leading to a selection of older sample when compared to the other fully adjusted models. Moreover, if the study question concerning binge drinking had been phrased separately for both genders allowing the use of a lower threshold value for women, a bigger proportion of women might have been defined as binge drinkers. Even though several covariates were used, the associations may also be affected by unmeasured confounders (e.g., chronic diseases [83, 84], mental health issues [85], temperament [86], transitions and life events [87], social support [88], or occupational status [87]). Conclusions on causality may be biased, as observational studies can include reverse causation. The study sample presents a Finnish population, and therefore, the results are not necessarily generalizable to other populations with, for example, a different socio-economic or ethnic background. Finally, researchers and readers need to acknowledge that trajectory group membership is not certain [89] as it only presents the probability of the participant to follow a trajectory.

In past studies, the previous PA level has been found to predict the future PA level [42, 90]. The current study adds to those findings by highlighting the importance of managing to increase LTPA during the life-course as not only persistent but also increasing activity was associated with several healthy behaviors in adulthood. This is an encouraging message for health promotion: the LTPA level in childhood and adolescence does not necessarily determine the LTPA level later in life, and LTPA initiated even after adolescence may play a role in adoption to other health-promoting behaviors in adulthood. These results may be leveraged as a platform for trajectory group-specific PA counseling with potential ramifications for additional healthy lifestyle choices. Our results also generate hypotheses for future qualitative research on the reasons underlying LTPA behavior, and for intervention studies to ascertain the causal relations behind these associations. Moreover, the constantly developing objective measurements enable collecting data on how PA, sedentary behavior, and sleeping integrate across the whole day (see e.g. [91]). Studies using accelerometers can provide a more comprehensive understanding of the codependency of these so-called time-use behaviors. Future longitudinal studies could identify trajectories/profiles of 24-h time-use behaviors (see e.g. [92]) and study their associations with other lifestyle choices.

## Conclusions

The present findings underline the different associations between life-course LTPA trajectories and various other health-related behaviors in adulthood and the sex differences in these associations. While the findings corroborated previous findings on the accumulation of healthy behaviors with persistent activity and of unhealthy behaviors with persistent inactivity and low activity, they also highlighted the meaning of increase in LTPA level. Especially, the study offered insight into the association between persistent LTPA during life-course and increased LTPA after childhood or adolescence with healthy eating and non-smoking in both genders and having less sleep difficulties in middle-aged women. The results suggest that to be physically active in adulthood does not necessarily require the initiation of a physically active lifestyle already in childhood. Promoting LTPA at all ages is important, not only because it may increase the level of PA, but because of its potential associations with healthy lifestyle choices later in life.

## Supplementary Information

**Additional file 1: Supplementary file 1.** Statistical analyses used for identifying the leisure-time physical activity trajectories and the description of the trajectories.

**Additional file 2: Supplementary file 2.** Description of the covariates.

**Additional file 3: Supplementary file 3.** STROBE-nut checklist.

**Additional file 4: Supplementary file 4.** Effect sizes (Cohen’s d and h) corresponding to adjusted models two and three for both genders.

## Data Availability

The datasets analyzed in this study are not publicly available for ethical and legal reasons but are available from the Publication Committee of the YFS on reasonable request. For more information on requests related to dataset access, please contact Professor Olli Raitakari, Project Director of the YFS, University of Turku, Finland, olli.raitakari@utu.fi.

## Abbreviations

BCH: Bolck-Croon-Hagenaars approach
BMI: Body mass index
ES: Effect size
LTPA: Leisure-time physical activity
PA: Physical activity
YFS: The Cardiovascular Risk in Young Finns Study
