# Supplementary material for: Life-course leisure-time physical activity trajectories in relation to health-related behaviors in adulthood: the Cardiovascular Risk in Young Finns study
Source: BMC Public Health. 2021 Mar 19;21:533. doi: 10.1186/s12889-021-10554-w (PMC7977567; doi:10.1186/s12889-021-10554-w)
Supplement: Supplementary file 1 — Additional file 1: Supplementary file 1. Statistical analyses used for identifying the leisure-time physical activity trajectories and the description of the trajectories. [file 12889_2021_10554_MOESM1_ESM.docx]

**Supplementary file 1.** Statistical analyses used for identifying the leisure-time physical activity trajectories and the description of the trajectories.

**Statistical analyses used to identify leisure-time physical activity trajectories**

Latent variable mixture modeling is a person-centered statistical tool that enables researchers to study similarities and differences between individuals in a data-driven manner [1–3]. The goal is to probabilistically assign study participants into subgroups by inferring each individual’s membership to latent classes [2]. In the current study, self-reported leisure-time physical activity (LTPA) subgroups were studied. Participants with at least one measurement of LTPA were included. The modeling enables including participants with only one measurement since the participant will have his/her own probabilities calculated for his/her membership in all of the latent classes estimated [2].

Latent profile analysis, a type of mixture modelling, was conducted separately for males and females to identify distinct LTPA trajectories from childhood to adulthood. Before modelling commenced, the longitudinal data were rearranged so that time points referred to ages instead of measurement years. Because the content of the LTPA questionnaires changed in 1992, overlapping items were recoded for missing values. After recoding, the LTPA questionnaires were similar in content from age 9 to 21 and from age 24 to 48. The latent profile analysis was based on the means of the LTPA outcome measures from age 9 to 48, and error variances were assumed to be equal across classes.

Models with one to six LTPA classes were fitted. Statistical criteria for the goodness-of-fit of each model was used to decide the number of classes. The criteria evaluated in this study were Akaike’s information criterion (AIC), the Bayesian information criterion (BIC) and the sample-size adjusted BIC (ABIC). For all three criteria, the lower the value, the better the fit of the model. In addition, the Vuong-Lo-Mendell-Rubin likelihood ratio test (VLMR), Lo-Mendell-Rubin adjusted likelihood ratio test (LMR) and parametric bootstrapped likelihood ratio test (BLRT) statistical tests were used to determine the optimum number of classes. The estimated model was compared to the model with one class less, a lower *p-*value indicating that the model with one class less is rejected in favour of the estimated model [1]. Entropy values and the average posterior probabilities (AvePP) for most likely latent class membership were used to evaluate the quality of the classification. Both measures ranged from 0 to 1, with the value 1 indicating a perfect classification. Values higher than 0.7 were considered acceptable for the average posterior probabilities [4, 5]. Missing data were assumed to be missing at random (MAR). Model parameters were estimated by using the full information maximum likelihood (FIML) method with robust standard errors, thereby enabling use of all the available data.

**Description of the leisure-time physical activity trajectories for males from childhood to adulthood**

The following four LTPA trajectory classes were identified for males: persistently low-active (41 %), decreasingly active (16 %), increasingly active (31 %), and persistently active (12 %) (Figure 1A). At each additional step in the modelling, the ABIC continued to decrease and the *p*-value of the BLRT remained low (<.001) (see Table 2 in [6]). However, in the five-class solution, the AvePP value for one of the latent classes fell below the acceptable level (<0.7), the *p*-values of the VLMR and LMR were higher than 0.05 and the BIC started to rise again, hence the four-class solution was considered optimal.

Males on the persistently active trajectory remained the most active (mean LTPA index value 11.6) while those on the persistently low-active trajectory remained the least active almost throughout the study period (mean LTPA index value 7.8). Among the low-active participants, LTPA decreased considerably between ages 12 (mean LTPA index value 9.2; 95% CI 8.9-9.4) and 21 (mean LTPA index value 7.1; 95% CI 6.8-7.3). A decline in LTPA for those on the decreasingly active trajectory also begun around age 12 (mean LTPA index value 11.5; 95% CI 11.2-11.8), continuing until age 48 (mean LTPA index value 7.5; 95% CI 6.3-8.7) when dropping to the level of the low-active participants (mean LTPA index value 7.9; 95% CI 7.4-8.4). At age 18, the LTPA level of the increasers reached its lowest point (mean LTPA index value 8.6; 95% CI 8.3-9.0), and at the age of 24 its highest point (mean LTPA index value 10.3; 95% CI 9.5-11.1). After the age of 24, the increasingly active trajectory remained rather stable. Despite that the trajectory was stable in adulthood, the trajectory was labelled as the increasingly active trajectory since it differed in shape from the other trajectories by being the only one where an increasing tendency of LTPA was observed after childhood and adolescence (Figure 1A).

**Description of the leisure-time physical activity trajectories for females from childhood to adulthood**

The following five LTPA trajectory classes were identified for females: persistently inactive (17 %), persistently low-active (52 %), decreasingly active (12 %), increasingly active (15 %), and persistently active (3 %) (Figure 1B). The *p*-values of the VLMR and LMR were below 0.05 only up to the three-class solution. However, since the ABIC continued to decrease substantially and the *p*-value of the BLRT was lower than 0.001 in each additional step, the modelling was continued up to the six-class solution (see Table 2 in [6]). At the six-class solution, the AvePP fell below the acceptable level (<0.7) in two of the latent classes, and the BIC started to increase again; hence, the five-class solution was considered optimal.

Among females, the biggest changes in LTPA level occurred before age 27, after which it stabilized. The increasingly active trajectory was an exception, showing a continuously increasing trend from childhood to adulthood: the mean LTPA index value increased from 8.5 (95% CI: 8.1-9.0) to 11.2 (95% CI: 9.9-12.5) between ages 9 and 48. The increasers were the second least active participants at age 9, but around age 40 they became the most active, exceeding the LTPA level of the persistently active participants. For those on the decreasingly active trajectory, the decline in LTPA started at age 18 (LTPA index value 10.9; 95% CI: 9.6-12.1) and continued to the end of the follow-up at age 48 (LTPA index value 8.4; 95% CI: 7.3-9.5). The trajectory was labelled as the decreasingly active one since these participants were comparatively active in childhood and adolescence until their LTPA decreased into the level of the low-active participants in adulthood. A decrease in LTPA was also seen among those on the low-active trajectory from ages 9 (LTPA index value 8.9; 95% CI: 8.6-9.2) to 18 (LTPA index value 7.8; 95% CI: 7.6-8.1), with slight increase in mid-adulthood. However, this decrease was smaller than that of the decreasingly active females. The participants on the persistently inactive trajectory remained the least active, showing a slightly decreasing tendency in LTPA across the study period (mean LTPA index value 7.2) (Figure 1B).

**Abbreviations**

ABIC: Adjusted Bayesian information criterion

AIC: Akaike’s information criterion

AvePP: Average posterior probabilities

BIC: Bayesian information criterion CI: Confidence interval

BLRT: Parametric bootstrapped likelihood ratio test

LMR: Lo-Mendell-Rubin adjusted likelihood ratio test

LTPA: Leisure-time physical activity

VLMR: Vuong-Lo-Mendell-Rubin likelihood ratio test

**References**

1. Muthén LK, Muthén BO. Mplus User’s Guide (1998-2017). 8th edition. Los Angeles, CA, USA; 2017.

2. Berlin KS, Parra GR, Williams N a. An introduction to latent variable mixture modeling (part 1): Overview and Cros-Sectional Latent Class and Latent Profile Analyses. J Pediatr Psychol. 2014;39:174–87. doi:10.1093/jpepsy/jst085.

3. Nagin DS. Group-based modeling of development. Cambridge Massachusetts: Harvard University Press. 2005.

4. Nylund-Gibson K, Choi AY. Ten Frequently Asked Questions about Latent Class Analysis. Transl Issues Psychol Sci. 2018. doi:10.1037/tps0000176.

5. Nagin DS. Group-based modeling of development. Cambridge, Mass.: Harvard University Press; 2005.

6. Lounassalo I, Hirvensalo M, Kankaanpää A, Tolvanen A, Palomäki S, Salin K, et al. Associations of leisure-time physical activity trajectories with fruit and vegetable consumption from childhood to adulthood: The cardiovascular risk in young finns study. Int J Environ Res Public Health. 2019;16:1–17.
