# Supplementary material for: Life-course leisure-time physical activity trajectories in relation to health-related behaviors in adulthood: the Cardiovascular Risk in Young Finns study
Source: BMC Public Health. 2021 Mar 19;21:533. doi: 10.1186/s12889-021-10554-w (PMC7977567; doi:10.1186/s12889-021-10554-w)
Supplement: Supplementary file 2 — Additional file 2: Supplementary file 2. Description of the covariates. [file 12889_2021_10554_MOESM2_ESM.docx]

**Supplementary file 2. Description of the covariates.**

Age, body mass index (BMI), level of education and marital status were elicited in 2011 and used as covariates. Weight was measured with a digital scale and height with wall-mounted stadiometer. BMI was calculated as kg/m^2^. Self-reported number of years of education was used as a proxy for socioeconomic status. Marital status was dichotomized: 1=unmarried, divorced, legally separated or widowed, and 2=married, in a registered relationship or cohabiting. Total energy intake was assessed based on the food frequency questionnaire in 2011 [4] and used as a covariate for dietary behavior in the analyses.

In addition, health behaviors in childhood, adolescence or young adulthood were used as covariates for the corresponding outcome health behaviors in adulthood. A healthy diet index was created from the 1989 data, with food items differing slightly from those in the 2011 index as the questionnaire had been modified and improved during the intervening years. Food items defined as healthy were fruits, vegetables, fish, and vegetable fats; unhealthy items were sausage dishes, sugared beverages, sweets, pastry and ice cream. Consumption frequencies of different food items per month were assessed on a scale from 1 to 6. For the healthy foods, 6=daily consumption of the item and 1=never or nearly never. For the unhealthy foods, the scale was reversed. The points were summed to create an index ranging from 9 to 54, with higher values indicating a healthier diet.

Finally, the following variables were used as covariates when studying the associations between the corresponding adulthood behaviors and LTPA trajectories: screen time assessed in 2001 (television and computer time in hours per day), smoking in 1989 (scale from 1=non-smoker to 4=regular smoker), total of binge drinking occasions in 1989 (scale from 1=none to 5=over 10 occasions), meeting the sleep recommendations (7-9 hours of sleep/night) in 1986 and feeling fatigue (scale from 1=rarely/never to 4=daily) in 1986. The participants were aged 12-27 years in 1989 and 24-39 years in 2001. Sleep duration and fatigue were assessed from a subsample of the 18-, 21- and 24-year-old participants in 1986.

The year 1989 was the first follow-up year when smoking and alcohol consumption were queried from all six age cohorts in the Cardiovascular Risk in Young Finns Study. In order to use as big sample as possible, data collected in 1989 concerning previous smoking and binge drinking was used. Also, data on dietary behavior was collected in 1989 and therefore was used as covariate. Screen time and sleeping were not queried in 1989. Screen time was queried in year 2001 for the first time and sleeping behavior in 1986 from a subsample and then again in 2001, 2007 and 2011 from the whole study sample. This is why data concerning previous sleeping behavior was from the year 1986 and screen time from 2001.

**References**

1. Poortinga W. The prevalence and clustering of four major lifestyle risk factors in an English adult population. Prev Med (Baltim). 2007;44:124–8.

2. Oftedal S, Vandelanotte C, Duncan MJ. Patterns of diet, physical activity, sitting and sleep are associated with socio-demographic, behavioural, and health-risk indicators in adults. Int J Environ Res Public Health. 2019;16:1–14.

3. Lahti-Koski M, Pirjo Pietinen MH, Vartiainen E. Associations of body mass index and obesity with physical activity, food choices, alcohol intake, and smoking in the 1982–1997 FINRISK Studies. Am J Clin Nutr. 2002;75:809–17.

4. Paalanen L, Männistö S, Virtanen MJ, Knekt P, Räsänen L, Montonen J, et al. Validity of a food frequency questionnaire varied by age and body mass index. J Clin Epidemiol. 2006;59:994–1001.

5. Telama R. Tracking of physical activity from childhood to adulthood: A review. Obes Facts. 2009;2:187–95.

6. te Velde SJ, Twisk JWR, Brug J. Tracking of fruit and vegetable consumption from adolescence into adulthood and its longitudinal association with overweight. Br J Nutr. 2007;98:431–8.

7. Paavola M, Vartiainen E, Haukkala A. Smoking, alcohol use, and physical activity: A 13-year longitudinal study ranging from adolescence into adulthood. J Adolesc Heal. 2004;35:238–44.
