# Supplementary material for: Life-course leisure-time physical activity trajectories in relation to health-related behaviors in adulthood: the Cardiovascular Risk in Young Finns study
Source: BMC Public Health. 2021 Mar 19;21:533. doi: 10.1186/s12889-021-10554-w (PMC7977567; doi:10.1186/s12889-021-10554-w)
Supplement: Supplementary file 4 — Additional file 4: Supplementary file 4. Effect sizes (Cohen’s d and h) corresponding to adjusted models two and three for both genders. [file 12889_2021_10554_MOESM4_ESM.docx]

**Supplementary file 4**. Effect sizes (Cohen’s d and h) corresponding to adjusted models two and three for both genders.

|  |  |  |  |  |  |  |  |  |  |  |  |  |  |  |  |
| --- | --- | --- | --- | --- | --- | --- | --- | --- | --- | --- | --- | --- | --- | --- | --- |
|  |  |  | Cohen’s d | | | | | | | | | |  | Cohen’s h | |
|  |  |  | Healthy diet | | Screen time | | Smoking | | Binge drinking | | Severity of sleep difficulties | |  | Probability of having recommended amount of sleep | |
|  | | | M2 | M3 | M2 | M3 | M2 | M3 | M2 | M3 | M2 | M3 |  | M2 | M3 |
| Comparison between LTPA trajectories, women: | | |  |  |  |  |  |  |  |  |  |  |  |  |  |
| Persistently inactive | vs. | Persist. low-active | *0.44* | 0.67 | (0.27) | (0.07) | *0.25* | (0.00) | (0.37) | (0.25) | *0.24* | (0.24) |  | (0.12) | (0.09) |
| Persistently inactive | vs. | Decreasingly active | 0.50 | 0.60 | (0.41) | (0.23) | *0.43* | (0.17) | (0.24) | (0.36) | *0.38* | 0.50 |  | (0.20) | (0.01) |
| Persistently inactive | vs. | Increasingly active | **1.00** | **1.08** | (0.10) | (0.00) | *0.48* | *0.38* | (0.53) | (0.54) | *0.39* | **0.91** |  | (0.06) | (0.10) |
| Persistently inactive | vs. | Persistently active | **1.08** | **1.08** | (0.32) | (0.21) | 0.50 | (0.25) | (0.46) | (0.46) | 0.64 | **1.14** |  | (0.22) | (0.11) |
| Persist. low-active | vs. | Decreasingly active | (0.05) | (0.08) | (0.25) | (0.25) | (0.18) | (0.18) | (0.14) | (0.14) | (0.13) | (0.25) |  | (0.32) | (0.08) |
| Persist. low-active | vs. | Increasingly active | 0.57 | *0.41* | (0.14) | (0.07) | (0.19) | *0.38* | (0.15) | (0.30) | (0.13) | 0.63 |  | (0.06) | (0.01) |
| Persist. low-active | vs. | Persistently active | 0.61 | (0.37) | (0.16) | (0.24) | (0.27) | (0.27) | (0.14) | (0.28) | *0.37* | **0.87** |  | (0.34) | (0.20) |
| Decreasingly active | vs. | Increasingly active | 0.52 | 0.50 | (0.32) | (0.25) | (0.00) | (0.24) | (0.37) | (0.19) | (0.00) | (0.42) |  | (0.26) | (0.09) |
| Decreasingly active | vs. | Persistently active | 0.59 | (0.48) | (0.09) | (0.00) | (0.11) | (0.11) | (0.35) | (0.18) | (0.29) | **0.73** |  | (0.02) | (0.12) |
| Increasingly active | vs. | Persistently active | (0.03) | (0.05) | (0.23) | (0.23) | (0.13) | (0.13) | (0.00) | (0.00) | (0.29) | (0.29) |  | (0.28) | (0.21) |
| Comparison between LTPA trajectories, men: | | |  |  |  |  |  |  |  |  |  |  |  |  |  |
| Persist. low-active | vs. | Decreasingly active | *0.40* | (0.29) | (0.21) | (0.26) | (0.22) | (0.14) | (0.08) | (0.01) | (0.24) | (0.56) |  | (0.06) | (0.11) |
| Persist. low-active | vs. | Increasingly active | **0.80** | 0.64 | *0.45* | 0.55 | *0.38* | *0.38* | (0.13) | (0.16) | (0.00) | (0.28) |  | (0.18) | (0.11) |
| Persist. low-active | vs. | Persistently active | 0.76 | 0.60 | (0.10) | (0.16) | *0.36* | *0.25* | (0.12) | (0.03) | (0.19) | (0.18) |  | (0.13) | (0.07) |
| Decreasingly active | vs. | Increasingly active | (0.40) | (0.35) | (0.24) | (0.29) | (0.14) | (0.24) | (0.22) | (0.18) | (0.24) | (0.30) |  | (0.24) | (0.00) |
| Decreasingly active | vs. | Persistently active | (0.37) | (0.31) | (0.12) | (0.11) | (0.15) | (0.12) | (0.04) | (0.04) | (0.41) | (0.72) |  | (0.20) | (0.04) |
| Increasingly active | vs. | Persistently active | (0.03) | (0.03) | *0.43* | *0.48* | (0.04) | (0.09) | (0.27) | (0.14) | (0.19) | (0.49) |  | (0.05) | (0.04) |
| Numbers in bold represent large magnitude of effect (≥0.80), underlined numbers represent medium magnitude of effect (≥0.50) and numbers in italic represent small magnitude of effect (≥0.20). The Cohen’s d and h values are marked in parentheses if there was no significant difference observed between the mean values of the health-related behaviors across the LTPA trajectory classes.  *M2* model 2; *M3* model 3; *LTPA* leisure-time physical activity; *vs.* versus; *Persist*. persistently | | | | | | | | | | | | | | | |
